# Overdetection, overtreatment and costs in prostate-specific antigen screening for prostate cancer

**DOI:** 10.1038/sj.bjc.6605422

**Published:** 2009-11-10

**Authors:** E A M Heijnsdijk, A der Kinderen, E M Wever, G Draisma, M J Roobol, H J de Koning

**Affiliations:** 1 Department of Public Health, Erasmus MC, University Medical Centre Rotterdam, PO Box 2040, 3000 CA Rotterdam, The Netherlands; 2 Department of Urology, Erasmus MC, University Medical Centre Rotterdam, PO Box 2040, 3000 CA Rotterdam, The Netherlands

**Keywords:** prostate cancer, screening, overdetection

## Abstract

**Background:**

Prostate cancer screening with prostate-specific antigen (PSA) has shown to reduce prostate cancer mortality in the European Randomised study of Screening for Prostate Cancer (ERSPC) trial. Overdetection and overtreatment are substantial unfavourable side effects with consequent healthcare costs. In this study the effects of introducing widespread PSA screening is evaluated.

**Methods:**

The MISCAN model was used to simulate prostate cancer growth and detection in a simulated cohort of 100 000 men (European standard population) over 25 years. PSA screening from age 55 to 70 or 75, with 1, 2 and 4-year-intervals is simulated. Number of diagnoses, PSA tests, biopsies, treatments, deaths and corresponding costs for 100 000 men and for United Kingdom and United States are compared.

**Results:**

Without screening 2378 men per 100 000 were predicted to be diagnosed with prostate cancer compared with 4956 men after screening at 4-year intervals. By introducing screening, the costs would increase with 100% to €60 695 000. Overdetection is related to 39% of total costs (€23 669 000). Screening until age 75 is relatively most expensive because of the costs of overtreatment.

**Conclusion:**

Introduction of PSA screening will increase total healthcare costs for prostate cancer substantially, of which the actual screening costs will be a small part.

Prostate cancer is one of the leading causes of cancer deaths in men. Early detection as a result of screening may result in a decrease in prostate cancer mortality as shown in the European Randomised study of Screening for Prostate Cancer (ERSPC) trial (Schröder *et al*, 2009). In this European trial, screening with prostate-specific antigen (PSA) reduced prostate cancer mortality by at least 20%.

Apart from the effects on mortality and the balance with unfavourable effects, the decision whether to introduce screening of prostate cancer will also depend on the expected costs of screening and treatment. The introduction of PSA screening will lead to a substantial increase in cancers detected. In the Rotterdam section of the ERSPC trial, the first round detection rate in the screened arm (54 cases per 1000 men) was nearly 30 times the incidence in 1991 without screening (2 cases per 1000) and 17 times the incidence in the control arm (3 cases per 1000) (Draisma *et al*, 2003; van der Cruijsen-Koeter *et al*, 2005). It is expected that part of the screen-detected prostate tumours (7–56%) might never give rise to clinical symptoms and might not lead to death caused by prostate cancer (Zappa *et al*, 1998; Draisma *et al*, 2003, 2009; Telesca *et al*, 2008). These overdetected, indolent prostate cancers are an important factor in determining the cost-effectiveness and desirability of a population-wide screening programme on prostate cancer. By using a simulation model (Draisma *et al*, 2003, 2006), the number of diagnoses, stage distribution and costs can be predicted for a future period using the screening algorithm as was done in the ERSPC trial.

The purpose of this study is to predict the costs of diagnosis, primary treatment of prostate cancer and the costs of overdetection when screening by PSA testing as done in the ERSPC trial is introduced population wide and to compare these with the costs of treatment when no screening is performed.

## Materials and methods

### Model description

The computer program MISCAN, MIcrosimulation SCreening ANalysis, was used for the evaluation of prostate cancer screening. MISCAN is a stochastic package simulating individual life histories. The MISCAN model reproduces age-specific incidence of prostate cancer as well as mortality from other causes than prostate cancer. A more detailed description of the basic MISCAN model can be found elsewhere (Draisma *et al*, 2003, 2006). The model has been updated with more recent data from the ERSPC trial Rotterdam.

A cohort of 100 000 men was used with an age distribution according to the European Standard Population 2003 (National Centre for Health Outcomes Development, NCHOD, UK). The probability that a man will develop prostate cancer is calculated using the disease and treatment-specific parameters estimated from the Rotterdam ERSPC trial data.

After invitation a PSA test will be performed. At a PSA level ⩾3 ng ml^−1^ a lateralised sextant biopsy is recommended. At a lower PSA level the screened individual will have to come back for a next screening after 4 years. The biopsy tissue is analysed by a pathologist. If the outcome is negative the man has to come back after 4 years. If positive, the individual has prostate cancer and he will be informed by his general practitioner. After staging, the individual will undergo primary therapy. Individuals with the disease in an advanced stadium (metastasis) will undergo palliative therapy. When primary therapy (radical prostatectomy, radiotherapy or active surveillance) is pursued the individual will, after follow-up, either be cured of prostate cancer or enter the advanced disease stadium and he will receive palliative therapy. It is also possible that cancer is detected between screening rounds. The men with interval cancers will follow the same routes after staging.

### Model application

Screening was modelled at ages 55–70 years with a 4-year interval (at ages 55, 59, 63 and 67 years) for the period 2008–2033 and a hypothetical screening attendance of 100%. Three alternative screening strategies have been modelled: (1) screening from age 55 to 70 with 1-year intervals (2) screening from age 55 to 70 with 2-year intervals and (3) screening from age 55 to 75 with 4-year intervals. The outputs of the model were the number of diagnoses by stage (T, Gleason score and metastasis) and age in the situation without screening and the number of screen visits, diagnoses by stage (T, Gleason score and metastasis), age, divided in clinically diagnosed (interval cancers), relevant (screen-detected cancers that would have given rise to clinical symptoms later in life) and overdetected (screen-detected cancers that would never given rise to clinical symptoms and would not have lead to death caused by prostate cancer) in the situation with screening (Draisma *et al*, 2009), all in the period 2008–2033.

To take false positive biopsies into account, the total number of biopsies of the screen-detected cancers was calculated using the predicted number of diagnoses and the mean positive predictive value of 22.3% (Postma *et al*, 2007) of a biopsy in the screen arm of the ERSPC trial. For the clinically detected cancers and the diagnosed cancers in the situation without screening the positive predictive value of 35.8% (Otto *et al*, 2003) of a biopsy in the control arm of the ERSPC trial was used.

The individuals with metastases are treated with palliative therapy. The individuals without metastases are treated with radical prostatectomy, radiotherapy or active surveillance. The distribution of these therapies were determined from the observed frequencies of the therapies in the screen arm and control arm of the ERSPC Rotterdam in the period 2000–2006, and were based on clinical T stage of the tumour, Gleason score and age of diagnosis.

The percentages of men that are cured were estimated by the 10-year relative survival by clinical T stage in 1989–2005 in the Netherlands. For T1 and T2, a relative survival of 87% was used, for T3 a relative survival of 77% (source: IKA, Comprehensive Cancer Centre Amsterdam).

### Costs

Direct medical costs were calculated, mainly based on Dutch sources, for the year 2008. The costs were obtained from literature, own estimates or tariffs from the Dutch Healthcare Authority 2008 (NZA), the governmental supervisor of healthcare budget in the Netherlands. Indirect costs are not included. All cost estimates are calculated in €2008. Corrections for inflation were made if necessary.

The unit costs of screening, diagnosis, primary therapy, follow-up and palliative therapy are presented in Table 1. The costs for invitation of screening, staging and follow-up are estimated, based on ERSPC data and expected diagnostics. The costs for radical prostatectomy and radiation therapy are obtained from literature (Malmberg *et al*, 1997; Perez *et al*, 1997; Burkhardt *et al*, 2002; Makhlouf *et al*, 2002) and own hospital data. To estimate the costs of active surveillance the protocol of the PRIAS study (van den Bergh *et al*, 2007) was used: During 7 years, 19 PSA tests, 10 digital rectal examination (DRE) and 4 biopsies are scheduled. Finally, the costs of advanced disease were based on chart review (Beemsterboer *et al*, 1999). The costs for palliative therapy consisted of costs for outpatient visit, assessment, treatment (hormonal, radiotherapy, surgery and other) and hospital days.

To give an indication what the results of this study would mean for the introduction of widespread PSA screening in a country, the costs of screening and treatment of prostate cancer were also calculated for the United Kingdom (30 million men) and the United States (305 million men). A healthcare-specific purchasing power parity, including exchange rate, of 0.743 for the United Kingdom and 1.137 for the United States was applied to the costs to account for relatively different healthcare costs in those countries compared with the Netherlands in 2008. The assumptions for the incidence and progression of the disease and the frequency of treatments remained the same.

## Results

The differences in number of men diagnosed in the situations without and with screening (4-year interval until age 70) per 100 000 men in 25 years are presented in Table 2. Without screening, 6642 biopsies are performed and 2378 men are diagnosed with prostate cancer. Radical prostatectomy and radiation therapy are the most frequently used treatments (both 30%), followed by active surveillance (18%) and 514 men receive palliative therapy (22%). After primary treatment, another 241 men receive palliative therapy as well in follow-up.

**Figure 1:**
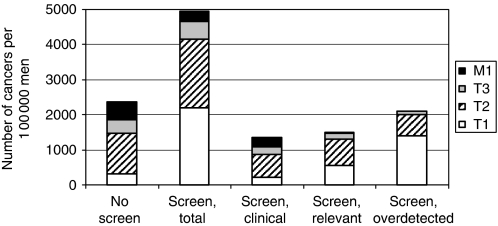
The number and stage distribution of cancers per 100 000 men in the next 25 years, in the situation without screening and the situation with screening (divided in clinically detected cancers, relevant cancers and overdetected cancers). The screening attendance is 100% for the ages 55–70 with a 4-year interval. In each column, the cancers are divided in stage T1, T2, T3 and metastasis (M1).

In the situation with screening, 126 888 PSA tests and 19 946 biopsies are performed and 4956 men are diagnosed. Of all screen-detected cancers, 30% are relevant and 42% are overdetected. Figure 1 shows the stage distribution of the cancers detected in the situations with and without screening.

Owing to the shift in stage distribution, different treatments are offered in the screen-detected situation. In total, radiation therapy (36%) is now the most used treatment, followed by radical prostatectomy (32%) and active surveillance (26%); however, the distribution of treatments differs between the clinically detected, relevant and overdetected cancers. Fewer men (301) receive palliative therapy than in the situation without screening. Most of these 301 men, 259 (86%) are clinically detected.

In the situation without screening, the costs for diagnosis and treatment in 25 years are € 30 284 000 per 100 000 men (Table 3), consisting of € 1 129 000 for biopsies and € 29 155 000 for treatment. After the introduction of screening, the costs for diagnosis and treatment increase to € 60 695 000 (100% increase). The costs are € 3 045 000 for screening tests, € 3 391 000 for biopsies and € 54 259 000 for treatment. The costs for palliative therapy will decrease from € 9 374 000 to € 7 029 000, a savings of 25%. In total, only 10% of the additional costs in the screening situation are attributed to the screening programme itself and €23 669 000 (39% of the total costs) can be attributed to overdetected cancers.

**Figure 2:**
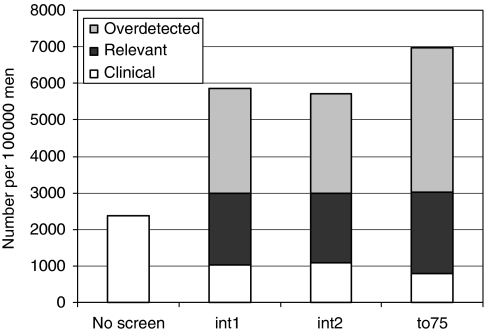
Number of cancers detected per 100 000 men in 25 years for three screening scenarios (1-year interval ages 55–70: int1, 2-year interval ages 55–70: int2, 4-year interval ages 55–75: to75) for clinically detected cancers (interval cancers), relevant cancers (screen-detected cancers that would have given rise to clinical symptoms later in life) and overdetected cancers (screen-detected cancers that would never given rise to clinical symptoms and would not lead to death caused by prostate cancer).

The results of the simulations of the three alternative screening strategies are shown in Table 4. Screening until age 75 detected the highest amount of cancers (6981, an increase of 41%, compared with screening until age 70). The amount of PSA tests increased with 29% to 163 545 and the number of biopsies with 50% to 29 954. Screening with a 1-year or a 2-year interval resulted in 453 740 (+258%) and 243 387 (+92%) PSA tests, 24 488 (+23%) and 23 759 (+19%) biopsies and a smaller increase in numbers of cancer detected of 5850 (+18%) and 5709 (+15%), respectively. Screening until age 75 resulted in the highest percentage of overdetected cancers (57%, which equals 3961 cancers). Both other strategies resulted in 49 and 48% overdetection (Figure 2).

All three alternative scenarios result in higher costs. Two-year interval screening is the less expensive scenario (70 million Euro), followed by 1-year interval screening (76 million Euro). Although the screening costs are lowest for the 4-year interval screening until age 75, the costs for treatment of overdetected cancers are much higher, resulting in increased total costs of 83 million Euro.

When screening (4-year interval until age 70) is introduced in the Unite Kingdom, about 800 000 extra cancers will be detected in 25 years, of which 630 000 will be overdetected. The costs for screening, diagnosis and treatment will be about 13.5 billion pound compared with 6.5 in the situation without screening. For the United States about 8 million extra cancers will be diagnosed, of which 6.4 million will be overdetected. The costs for screening, diagnosis and treatment will be 210 billion US dollars compared with 105 in the situation without screening.

## Discussion

PSA screening for prostate cancer has long been controversial. Although the PSA test is simple, safe and has an acceptable sensitivity and specificity, estimates of the costs, risk of overdiagnosis and side effects of unnecessary treatment are unfavourable. In our simulation, in the period 2008–2033, 2578 men extra per 100 000 men will be diagnosed with prostate cancer of which an estimated number of 2102 men will be overdiagnosed, accounting for 39% of the total costs for screening and treatment. Therefore, introduction of PSA population screening for prostate cancer for men aged 55–67 with 4-year intervals will increase the total costs of prostate cancer in the next 25 years significantly. The actual screening costs will only be a small part of the increase (10%). A shorter screen interval results in more cancers detected, more overdiagnosis and higher costs. Extended screening to age 75 results in even more cancers detected, more overdiagnosis and more costs. As the life years gained will probably be less for the older ages, it seems more appropriate to shorten the screen interval than to extend screening to age 75. However, quality of life and cost-effectiveness should also be taken into account.

The percentages of overdetected cancers found in this study, 42% and 57%, are comparable to what has been found earlier: 44–56% (Zappa *et al*, 1998; Draisma *et al*, 2003, 2009; Telesca *et al*, 2008). Telesca *et al* (2008) found lower percentages of 7–32%, depending on age and race, for the population of the United States. However, they used a higher PSA threshold for biopsy and a lower biopsy compliance. The risk of overdiagnosis is higher for prostate cancer as compared to other types of cancer, because prostate cancer shows a particularly high prevalence of latent lesions and because of the high age, the risk of death from other causes is higher (Zappa *et al*, 1998). In addition, quality of life aspects are not included. It was shown that even 5–10 years after the treatment, 23–48% of the prostate cancer survivors have urinary problems, 5–14% has bowel problems and 40–74% has sexual problems (Mols *et al*, 2009). These percentages were significantly higher than found in the age-matched normative population. Especially the overdetected cases will experience the negative consequences, without the benefits of screening.

There are few recent cost-effectiveness studies for PSA screening and results are ambiguous (Crawford and Abrahamsson, 2008). Less recent studies should be used with caution, because of changing treatments and changing incidence and mortality rates. In an overview of cost-effectiveness studies of prostate cancer screening, it was argued that the sensitivity, specificity and positive predictive value of the PSA test are acceptable (Crawford and Abrahamsson, 2008).

A hospital in the United Kingdom has measured the costs of the extra patients, referred to the hospital as a result of PSA screening by a recruitment company (Chelladurai *et al*, 2008). In 1 year, 80 additional patients at the costs of 250 000 £ (about € 4000 per patient) were treated. The authors state that these costs are likely to be an underestimation, because costs for further follow-up and treatment after 1 year have not been considered. In our study, the costs per additional patient detected would be about € 9000 in 1 year.

Several assumptions had to be made in the simulations. Most of the disease-specific parameters in the model have been fit to data of the ERSPC Rotterdam. The predicted clinical T stage of the tumours in the situation without screening compared well with the observed distribution in a part of the Netherlands in 1999–2001: T1 and T2: 62%; T3: 16%; T4: 22% in the simulation, compared with 56%, 21% and 22%, respectively, in the Netherlands (Visser and van Leeuwen, 2005). The treatments the diagnosed men received in our simulation were also based on the observed treatments in the ERSPC trial. These treatments can differ in the future. When active surveillance will be applied for more patients, the total treatment costs will be lower, whereas an increase in, for example, hormonal treatment or brachytherapy will increase the total costs. Recently, two nomograms have been validated that can help in the prediction of indolent cancers, and therefore reduce the number of overtreated cancers (Dong *et al*, 2008). Measures of uncertainty in the results are difficult to obtain, because there are many parameters in the model. Changes in natural history of the tumors would have an influence on the proportion of overdiagnosis. In the publication of Draisma *et al* (2003), the 95% confidence interval for proportion of overdetection was 46–57%, which means that despite the uncertainty, the costs for overdetection are always substantial.

In the model the effects of screening with 100% attendance is compared with a situation without screening. However, in most European countries, screening is already going on and when screening will be implemented less than 100% of men invited will participate. In the ERSPC Rotterdam section, it was estimated that 15% of the men in the control arm did have at least one PSA test for screening purposes (Roobol *et al*, 2009). As the population who attends opportunistic screening is not comparable with the general population (the ‘healthy screenee’ effect), we have chosen to compare all strategies with a situation of no screening at all. In addition, the attendance rate will not be 100% when screening is implemented. Comparing the strategy screening at ages 55–70 with 4-year intervals with an attendance of 80% with a background screening of 15% results in overdiagnosis of 1252 men. The total costs for the situation with 15% screening are 38 million Euro and with 80% screening 57 million Euro and therefore the increase in costs after implementing screening will be 50%.

A limitation of this study is that indirect costs (administrative costs, patient's loss of income from time off from work, travelling costs and time, costs of complications resulting from biopsy or treatment) are not included. Therefore, it is expected that the actual total costs of prostate cancer in a situation with screening will be higher than predicted in this study. However, even without these additional costs, the costs of overdetection are already substantial.

The costs for PSA test, DRE, TRUS and biopsy used in this study are comparable with the costs mentioned in a literature overview (Ekwueme *et al*, 2007). The pooled baseline resource costs extracted from 13 studies in Canada, Sweden, United Kingdom, Australia and Japan, calculated in US $ 2003 were $ 30.9 for PSA test, $ 33.5 for DRE, $ 103.8 for TRUS and $ 165 for biopsy (in 2008 € these costs would be: € 28.7, € 31.1, € 96.2 and € 152.3, respectively). These costs are comparable with the costs used in our study. In addition, the total predicted costs for prostate cancer without screening are comparable with the actual costs in the Netherlands. The costs of 30 million Euro per 100 000 men in 25 years can be recalculated as 96 million Euro per 8 million (all men in the Netherlands) per year. In 2005, the actual costs for prostate cancer in the Netherlands were 101.6 million Euro (Poos *et al*, 2008).

In conclusion, the implementation of PSA screening for prostate cancer will lead to a doubling of the total costs for prostate cancer. Only a small part of this increase can be attributed to the costs of screening itself. Most of the additional costs are for diagnosis and treatment, especially for overdiagnosed cases. Prevention of overdiagnosis and overtreatment, quality of life and cost-effectiveness should be considered in the discussion whether it is desirable to introduce widespread screening of prostate cancer.

## Figures and Tables

**Table 1:** Costs of prostate cancer screening, diagnosis, primary therapy and advanced disease in Euro (2008)

**Intervention**	**Costs**	**Source**
*Screening*	24	
Invitation	2	Estimation
Blood sample taking	9.5	NZA
PSA determination	12.5	NZA
		
*Diagnosis*	170	
Biopsy	92	NZA
PA research	33	NZA
GP consulting	45	20 min (tariff per hour € 135.5)
		
*Primary therapy*		
Staging	200	Estimation
Radical prostatectomy	11 800	Hospital data/literature (Burkhardt *et al*, 2002; Makhlouf *et al*, 2002)
Radiotherapy	14 178	Literature (Malmberg *et al*, 1997; Perez *et al*, 1997)
Active surveillance	1588	van den Bergh *et al* (2007)
19 PSA tests	418	
10 DRE	490	Estimation € 26 per test and 10 min (tariff per hour € 135.5)
Four biopsies	680	
Follow-up	150	Estimation
		
*Advanced disease*		
Palliative therapy	12 276	Literature (Beemsterboer *et al*, 1999)

Abbreviations: PSA=prostate-specific antigen; DRE=digital rectal examination.

**Table 2:** Number of prostate-specific antigen (PSA) tests, prostate cancer diagnoses and treatments of a cohort of 100 000 European Standard men of all ages in the period 2008–2033

		**Screening**			
	**No screening**	**Total**	**Clinical^a^**	**Relevant^a^**	**Overdetected^a^**
PSA tests	0	126 888	23 919 (19%)	43 014 (34%)	59 955 (47%)
Biopsies	6642	19 946	3760 (19%)	6761 (34%)	9424 (47%)
Cancers detected	2378	4956	1346 (27%)	1508 (30%)	2102 (42%)
Active surveillance	438	1310	266 (20%)	388 (30%)	656 (50%)
Radical prostatectomy	716	1559	410 (26%)	499 (32%)	651 (42%)
Radiation therapy	708	1786	412 (23%)	579 (32%)	795 (45%)
Palliative therapy	514	301	259 (86%)	42 (14%)	0 (0%)
Palliative therapy after primary treatment	241	267	133 (50%)	134 (50%)	0 (0%)

The screening attendance is 100% for the ages 55–70 with a 4-year interval.

a Cancers detected in the situation with screening are divided in clinically detected cancers (interval cancers), relevant cancers (screen-detected cancers that would have given rise to clinical symptoms later in life) and overdetected cancers (screen-detected cancers that would never given rise to clinical symptoms and would not lead to death caused by prostate cancer).

**Table 3:** Total costs of screening (ages 55–70 with a 4-year interval) and treatment of a cohort of 100 000 men of all ages in the period 2008–2033 in kEuro (2008)

		**Screening**			
	**No screening**	**Total**	**Clinical^a^**	**Relevant^a^**	**Overdetected^a^**
PSA tests	0	3045	574 (19%)	1032 (34%)	1439 (47%)
Biopsies	1129	3391	639 (19%)	1149 (34%)	1602 (47%)
Active surveillance	784	2342	475 (20%)	694 (30%)	1173 (50%)
Radical prostatectomy	8704	18 947	4976 (26%)	6066 (32%)	7906 (42%)
Radiation therapy	10 293	25 942	5987 (23%)	8405 (32%)	11 550 (45%)
Palliative therapy	6417	3751	3227 (86%)	524 (14%)	0 (0%)
Palliative therapy after primary treatment	2957	3277	1634 (50%)	1643 (50%)	0 (0%)
Total costs	30 284	60 695	17 512 (29%)	19 513 (32%)	23 669 (39%)

Abbreviation: PSA=prostate-specific antigen.

a Cancers detected in the situation with screening are divided in clinically detected cancers (interval cancers), relevant cancers (screen-detected cancers that would have given rise to clinical symptoms later in life) and overdetected cancers (screen-detected cancers that would never given rise to clinical symptoms and would not lead to death caused by prostate cancer).

**Table 4:** Number of PSA tests, prostate cancer diagnoses and treatments and costs (in kEuro 2008) of a cohort of 100 000 men of all ages in the period 2008–2033

	**55–70, 1 year**	**55–70, 2 year**	**55–75, 4 year**
PSA tests	453 740	243 387	163 545
Biopsies	24 488	23 759	29 954
Cancers detected	5850	5709	6981
Active surveillance	1714	1614	1942
Radical prostatectomy	1792	1779	2214
Radiation therapy	2099	2065	2608
Palliative therapy	245	251	217
Palliative therapy after primary treatment	218	246	277
Costs screening (kEuro)	10 890 (14%)	5841 (8%)	3925 (5%)
			
*Costs biopsies and treatment*			
Clinical	13 032 (17%)	13 695 (19%)	9889 (12%)
Relevant	22 758 (30%)	22 476 (32%)	27 412 (33%)
Overdetected	29 446 (39%)	28 524 (40%)	42 165 (51%)
Total costs (kEuro)	76 126	70 536	83 391

Abbreviation: PSA=prostate-specific antigen. Three screening scenarios are shown: screening for the ages 55–70 with a 1-year interval and a 2-year interval and screening for the ages 55–75 with a 4-year interval. The attendance is 100% in all scenarios.
